# Implementation of a Shared Decision-Making Tool for Osteoarthritis Treatment to Reduce Decisional Conflict

**DOI:** 10.1093/geroni/igab046.703

**Published:** 2021-12-17

**Authors:** Yashika Watkins, Rose Gonzalez, Charla Johnson, Ravneet Kaur

**Affiliations:** 1 Chicago State University, Cliffside Park, New Jersey, United States; 2 Movement is Life, Lake Ridge, Virginia, United States; 3 Franciscan Missionaries of Our Lady Health System, Baton Rouge, Louisiana, United States; 4 University of Illinois at Chicago, Crown Point, Indiana, United States

## Abstract

Shared decision making is a key component of patient centered care where clinical evidence and the patient’s preference and values are considered. Physical activity and weight loss are often recommendations in the treatment plan, especially in mild to moderate stage of osteoarthritis (OA). Movement is Life (MIL) created an innovative SDM tool to provide a framework for patient-centered discussions. The tool leverages an underlying Markov Model and represents the likely pain, activity levels, and lost productivity at three future time points. By comparing the patient’s likely progression depending on treatment choices compared to doing nothing, the patient has an illustration of future state. A pilot of N=108 women, ages 45-64, with chronic knee pain for at least three months and at least one co-morbidity (obesity, hypertension, diabetes) were randomized to a control (n=54) or intervention (n=54) arm of the study at eight centers across the United States. Results showed the demographic profiles were similar between the groups. At one-month, n=47 control and n=50 intervention patients returned for evaluation. Self-reported level of physical activity increased in the intervention group (56% vs 34%, p = 0.0229). Qualitative feedback from the intervention group indicated high satisfaction with use of the tool. Both groups reported a high likeliness to recommend the provider to a friend or family member. Inclusion of the SDM tool added an average of one minute to the patient counseling time over the control group. The quasi script provides a consistent communication pathway and may reduce disparities by addressing unconscious bias.

